# New treatment of pyoderma gangrenosum and hidradenitis suppurativa: A review

**DOI:** 10.1111/1346-8138.17031

**Published:** 2023-11-27

**Authors:** Keiichi Yamanaka

**Affiliations:** ^1^ Department of Dermatology Mie University Graduate School of Medicine Tsu Japan

**Keywords:** hidradenitis suppurativa, inflammatory skin disease, keratinocyte, neutrophil, pyoderma gangrenosum

## Abstract

Pyoderma gangrenosum (PG) and hidradenitis suppurativa (HS) are stubborn inflammatory skin diseases categorized as neutrophilic hypodermal dermatoses. These conditions exhibit connections with other autoinflammatory disorders driven by immune responses. Their pathogenesis is complex, rooted in significant imbalances in both innate and adaptive immune systems, particularly featuring elevated levels of tumor necrosis factor‐α (TNF‐α), interleukin (IL)‐1, IL‐8, IL‐17, and IL‐23. Studies involving skin tissue pathology and serology have indicated that targeting specific cytokines can bring therapeutic benefits. Indeed, many patients in clinical settings have responded positively to such interventions. Yet, given the diverse cytokines in play, focusing on a single one with antibody therapy might not always be effective. When resistance to biologics emerges, a combined approach targeting multiple overactive cytokines with immunosuppressants, for example cyclosporine and Janus kinase inhibitors, could be an option. In the current review, we explore recent therapeutic developments for PG and HS.

## 1 INTRODUCTION

### 1.1 Pyoderma gangrenosum

Pyoderma gangrenosum (PG) is a relatively rare inflammatory skin disease, classified under the umbrella of neutrophilic hypodermal dermatoses.^1^ Clinically, it manifests as painful, rapidly progressing skin ulcers distinguished by their undermined, irregular borders, and peripheral erythema.^2^, ^3^, ^4^ Globally, the estimated incidence of PG is between 3 and 10 cases per million individuals annually.^3^, ^5^ PG indicates an autoinflammatory disorder, frequently correlating with other immune‐mediated systemic diseases. The most common among these are inflammatory bowel disease, rheumatoid arthritis, and hematological malignancies. Moreover, PG can also be a feature of autoinflammatory syndromes such as pyogenic arthritis, PG, and acne (PAPA), PG, acne, and hidradenitis suppurativa (PASH), and pyogenic arthritis, PG, acne, and hidradenitis suppurativa (PAPASH).

The pathogenesis of PG is multifaceted, stemming from significant dysregulation of both innate and adaptive immunity in genetically susceptible individuals.^6^ An emerging understanding points to the follicular unit^7^ and, in cases involving the palms or soles, likely the sweat gland unit, as primary sites of initial impact. Such triggers precipitate a shift towards type 1/type 3‐biased inflammation^8^ and heightened inflammasome activation in those predisposed.^5^ This cascade culminates in a dysregulated environment dominated by neutrophils, marked by elevated concentrations of tumor necrosis factor‐α (TNF‐α), interleukin (IL)‐1α, IL‐1β, IL‐8, IL‐12, IL‐15, IL‐17, IL‐23, and IL‐36.^9^, ^10^, ^11^, ^12^, ^13^

Given the multifaceted array of cytokines involved, targeting a single cytokine with antibody therapy may not always yield optimal results. In instances of resistance to biological treatments, systemic suppression of the overexpressed cytokines might prove beneficial. An effective therapeutic approach generally starts with rapid‐acting immunosuppressants, such as corticosteroids and cyclosporine, to curb inflammation. This can be followed by the introduction of slower‐acting immunosuppressants that have more favorable safety profiles including biologics.^5^ While ulcerative lesions tend to require an extended period for epithelization, it is advisable to periodically evaluate the efficacy of treatments, with intervals reaching up to a month.^14^ Once immune suppression has been successfully achieved, surgical interventions, including vacuum‐assisted closure and skin grafting, emerge as effective solutions, particularly for extensive skin ulcers.^15^

### 1.2 Hidradenitis suppurativa

Hidradenitis suppurativa (HS), also called acne inversa or Verneuil's disease, is a chronic and debilitating inflammatory skin condition. Characterized by recurrent subcutaneous painful nodules, it often advances to persistent abscesses.^16^ Typically emerging post‐puberty, the condition exhibits deep‐seated, inflamed lesions predominantly in areas where apocrine glands are abundant, such as the axillary, inguinal, perineal, and anogenital regions. The course of HS is punctuated by cycles of remission and flare‐ups; without consistent treatment, these episodes become progressively severe. The signature abscesses and deep lesions of HS frequently evolve into draining sinus tracts. Over time, due to dermal fibrosis and subsequent healing, these tracts can induce skin deformities and contractures^16^, ^17^ HS has a notable prevalence, with estimates suggesting it affects 1%–4% of the Western population.^18^

The exact pathogenesis of HS remains elusive. However, it is believed that hyperkeratosis within the follicular epithelium, coupled with follicular occlusion, prompts the rupture of the hair follicle infundibulum, subsequently inciting dermal inflammation.^16^, ^19^ This inflammation can then pave the way for bacterial infiltration and secondary infections.^20^ Notably, there is a discernible link between HS and other immune‐mediated conditions, including Crohn's disease and PG. Current consensus increasingly acknowledges the significance of immune dysregulation in exacerbating the inflammation associated with HS.^16^, ^21^, ^22^ There appears to be a notable inclination towards the IL‐1 pathway and type 1 immune response in the context of HS. A range of cytokines, including TNF‐α, IL‐1β, IL‐17, and IL‐23, play roles in this immune dysregulation. In managing HS, beyond the standard usage of oral corticosteroids and immunosuppressive agents like cyclosporin, there is growing evidence supporting the efficacy of TNF‐α inhibitors as primary therapeutic agents.^22^, ^23^, ^24^ Additionally, IL‐1β is implicated in the inflammatory process, marked by the activation of caspase‐1, the inflammasome, and Nucleotide‐binding oligomerization domain‐like receptor family, pyrin domain‐containing 3 (NALP3). The early appearance of immune cells in HS‐affected skin, even before the emergence of an inflammatory lesion, underscores the pivotal role of the immune system in HS pathogenesis.^21^

In this review, we delve into the latest treatment advancements for PG and HS. While we scrutinize the roles of target molecules in the pathogenesis of both conditions, it is noteworthy that most of the available literature either offers limited clinical evidence or pertains to ongoing clinical trials.

## 2 NEWLY TREATMENT OPTIONS FOR PG AND HS

### 2.1 Biologics

#### 2.1.1 TNF‐α inhibitors

TNF‐α plays a pivotal role as a multifaceted proinflammatory cytokine, enhancing the activity of IL‐1β, IL‐6, and IL‐8. This, in turn, draws neutrophils and collaborates with TNF‐α itself to intensify the inflammatory state.^25^ Therapeutics like infliximab, adalimumab, etanercept, certolizumab pegol, and golimumab have gained global utilization. Notably, etanercept targets only the soluble form of TNF‐α. Meanwhile, infliximab, adalimumab, and golimumab engage both soluble and membrane‐bound TNF‐α forms, prompting the apoptosis of cells expressing TNF‐α. Distinctively, certolizumab, lacking an Fc domain, is unable to trigger apoptosis or induce complement‐dependent or antibody‐dependent cell‐mediated cytotoxicity.^26^

##### Pyoderma gangrenosum

Elevated levels of TNF‐α and its receptors have been observed in PG‐afflicted skin.^5^, ^27^ A burgeoning body of evidence advocates for the prioritization of TNF‐α inhibitors, especially infliximab and adalimumab, as primary treatment modalities.^26^, ^28^, ^29^ The effectiveness of infliximab in addressing PG has been validated in a randomized, double‐blinded controlled trial.^30^ Furthermore, anti‐TNF‐α inhibitors present as viable alternatives to mitigate the prolonged adverse effects associated with corticosteroids and cyclosporine.

##### Hidradenitis suppurativa

TNF‐α is believed to be at the forefront of driving the inflammatory cascade. Notably, its concentration in both serum and HS‐affected skin is markedly elevated compared to healthy individuals, coinciding with raised levels of IL‐1β, IL‐10, and IL‐17.^21^, ^31^, ^32^ While a diverse array of inflammatory cytokines is associated with the pathogenesis of HS, anti‐TNF‐α treatments appear to effectively dampen the presence of most of these proinflammatory agents.^33^, ^34^

#### 2.1.2 Adalimumab

##### Pyoderma gangrenosum

Adalimumab (ADA) is a human‐derived monoclonal antibody that targets both soluble and membrane‐bound TNF‐α. Through inhibiting the actions of TNF‐α, ADA enhances the innate immune response by diminishing the concentrations of pro‐inflammatory cytokines and inflammatory white blood cells.^17^, ^24^ Notably, ADA stands out as the sole treatment for HS endorsed by the US Food and Drug Administration (FDA). A recent review revealed that 75% of patients on adalimumab experienced full resolution of PG.^35^

##### Hidradenitis suppurativa

Compared to a placebo, ADA has demonstrated significant superiority in reducing pain, mitigating disease severity, and decreasing the count of skin lesions. Moreover, patients reported enhanced quality of life, alleviated pain intensity, and fewer depressive symptoms.^24^ ADA remains the sole therapy for HS endorsed by the US FDA. In phase III trials, fewer than 45% of participants experienced a 50% reduction in HS lesions by the 12th week when treated with ADA.^36^

#### 2.1.3 Infliximab

##### Pyoderma gangrenosum

Infliximab (IFX) is an IgG1 class monoclonal antibody that targets both soluble and membrane‐bound TNF‐α. Its efficacy in treating PG was confirmed in a randomized, double‐blind trial.^30^ Additionally, a phase III, open‐label study spanning multiple centers evaluated adalimumab's effectiveness and safety for patients with refractory PG in Japan. By the conclusion of the 26‐week treatment, 55% of the participants achieved complete healing.^37^

##### Hidradenitis suppurativa

IFX has demonstrated efficacy in enhancing the quality of life for HS patients.^17^ Previous research assessed the effectiveness and safety of IFX, revealing significant improvements in skin lesions.^24^, ^38^, ^39^

#### 2.1.4 Etanercept

##### Pyoderma gangrenosum

Etanercept (ETA) is a recombinant fusion protein that targets TNF‐α. Notably, ETA inhibits only the transmembrane form of TNF‐α, unlike other treatments that target both soluble and transmembrane forms.^17^, ^24^ In previous studies, etanercept led to complete healing in 53%–61% of patients.^35^, ^40^ ADA and IFX have demonstrated higher complete response rates compared to ETA.^40^

##### Hidradenitis suppurativa

Several trials have evaluated the efficacy of ETA in treating HS. However, these studies did not observe any notable differences in patient or physician global assessments, or in quality of life when comparing ETA to a placebo.^24^, ^41^

#### 2.1.5 Interleukin‐1α/β inhibitors: Anakinra and canakinumab

##### Pyoderma gangrenosum

Interleukin‐1α, constitutively expressed in keratinocytes, is released on cellular damage and plays a role in autoinflammatory diseases.^42^ This IL‐1α production regulates the release of factors such as granulocyte colony‐stimulating factor, IL‐6, CXCL‐1, and CXCL‐8, all of which play a role in neutrophil recruitment and activation. IL‐1β, found in macrophages, T lymphocytes, endothelial cells, fibroblasts, and activated keratinocytes, has elevated levels in PG lesions.^12^, ^13^ Once produced, the inactive pro‐IL‐1β undergoes cleavage by caspase‐1 to form its mature variant, which is then released. Notably, in PAPA syndrome, mutations in the PSTPIP1 gene lead to increased binding affinity to pyrin, facilitating inflammasome assembly. Within PG, IL‐1β amplifies the release of proinflammatory cytokines and chemokines, further influencing neutrophil activity.^26^

For PG treatment, IL‐1 inhibitors such as anakinra (which competitively blocks IL‐1α and IL‐1β), canakinumab, and gevokizumab (both targeting IL‐1β) have been used. Bermekimab (RA‐18C3), an IL‐1α inhibitor, is under exploration for treating PG.^35^ Current use of these inhibitors is mainly documented in case reports and phase II open‐label trials, where they have demonstrated potential benefits,^35^ Specifically, anakinra has been associated with marked clinical improvement, with recent studies reporting response rates of 59% (17/29) and full remission rates of 38% (11/29).^43^ Meanwhile, canakinumab showed a response in 64% (7/11) of cases and complete resolution in 55% (6/11) of cases, although some showed no response.^11^, ^35^, ^43^

##### Hidradenitis suppurativa

IL‐1β is a recognized marker of inflammation in HS. Given the immunomodulatory properties of anakinra, it emerges as a potential therapeutic alternative for HS patients who exhibit limited response to TNF‐α antagonists. Both prospective open‐label studies and comprehensive randomized double‐blind, placebo‐controlled trials have underscored the potential efficacy of anakinra, although a minority of patients remained unresponsive to treatment.^24^ Among other biological treatments, anti‐IL‐1 therapies have shown promise in preliminary studies.^44^, ^45^ In a double‐blind, randomized, placebo‐controlled phase II clinical trial involving 20 patients, the anakinra‐treated cohort showed a significant reduction in disease activity scores after 12 weeks compared to the placebo group (78% vs. 20%, *P* = 0.02). Furthermore, 78% achieved HiSCR by week 12, as opposed to 30% in the placebo group (*P* = 0.04). However, by the 24‐week mark, the difference in Hidradenitis Supprativa Clinical Response (HiSCR) achievement was no longer statistically significant (10% vs. 33%, *P* = 0.28).^44^, ^46^

#### 2.1.6 Interleukin‐17 inhibitors

##### Pyoderma gangrenosum

IL‐17 and its receptor exhibit pronounced expression in PG lesional skin.^7^, ^13^ IL‐17 promotes the release of chemokines that draw neutrophils and elicits the secretion of IL‐6, GM‐CSF, and IL‐8. Furthermore, Th17 cell counts are elevated in PG.^7^, ^13^, ^47^ A few accounts are highlighting the successful treatment of PG with IL‐17 inhibitors. A phase I–II pilot study sought to delve deeper into the therapeutic potential and safety of secukinumab monotherapy for PG. Within this study, seven patients were administered 300 mg of secukinumab weekly from weeks 0 to 4. Subsequently, two patients received doses every 4 weeks up to week 16, while the remaining five were dosed every 2 weeks until week 32. Of these, two exhibited significant reductions in ulcer dimensions, inflammation indicators, and scores on the Dermatological Life Quality Index by week 32. Universally, participants reported alleviated pain. Brodalumab, a human anti‐IL‐17 receptor A (IL‐17RA) monoclonal antibody, given at 210 mg either weekly or biweekly, led to improvement in three out of the assessed patients.^48^ It is worth noting that IL‐17 inhibition, and even transitioning between different IL‐17 inhibitors, has been associated with inducing PG in some cases.^26^, ^49^, ^50^, ^51^ This suggests that type 3 blockade might pave the way for a type 1 dominant environment.

##### Hidradenitis suppurativa

Levels of IL‐17A are notably elevated in both the bloodstream and lesional skin of HS patients.^21^ IL‐17A stimulates neutrophils and lymphocytes, and triggers the expression of other pro‐inflammatory cytokines, including IL‐1β, IL‐6, and TNF‐α.^21^ Secukinumab binds to IL‐17A, counteracting this inflammatory pathway, suggesting its potential therapeutic use in HS, although data from cases remain sparse.^24^ The effectiveness of secukinumab for treating moderate‐to‐severe HS was explored in the open‐label SUNRISE trial.^52^ Participants received secukinumab at two dosing schedules: an initial five 300 mg injections weekly, followed either by 300 mg every 4 weeks or every 2 weeks. By weeks 12 and 14, 65% and 70% of participants, respectively, achieved HiSCR.^41^ Brodalumab has demonstrated efficacy in reducing inflammation and enhancing psoriasis‐, keratinocyte‐, and neutrophil‐associated pathways in moderate‐to‐severe HS cases.^53^ Administered at 210 mg at weeks 0, 1, and 2, and biweekly thereafter, all 10 treated patients achieved HiSCR by week 2 and saw a 75% decline in abscess and nodule counts by week 24. Furthermore, 80% of these patients reached the International Hidradenitis Supprativa Severity Score System (IHS4) category shift by week 12. Other significant improvements were recorded in variables such as Visual Analog Scale (VAS) of pain, itch, global disease assessment, Dermatology Life Quality Index (DLQI), and the patient health questionnaire‐9, all measured from baseline at week 12.^41^ Currently, the potential of anti‐IL‐17A treatments for HS is a topic of investigation.^54^ A meta‐analysis identified bimekizumab as a promising biologic for the treatment of moderate‐to‐severe HS, particularly between weeks 12 and 16.^41^

#### 2.1.7 Interleukin‐23 inhibitors and ustekinumab

##### Pyoderma gangrenosum

IL‐23 is notably upregulated in PG skin lesions. IL‐23 plays a pivotal role in promoting and sustaining Th17 cells, which in turn produce IL‐17, amplifying neutrophil recruitment. The therapeutic arsenal against IL‐23 includes ustekinumab, which inhibits the p40 subunit of both IL‐12 and IL‐23, and agents like tildrakizumab, guselkumab, and risankizumab that specifically target the p19 subunit of IL‐23. A recent systematic review highlighted that 71% of patients administered with ustekinumab achieved a complete response.^43^ Moreover, an updated compilation of case studies focused on the efficacy of ustekinumab against PG indicated that 68% of patients witnessed total ulcer healing, while the remaining 32% observed partial amelioration.^55^ Periodic administrations of ustekinumab (90 mg every 2–3 months) have demonstrated improvements across various PG subtypes.^55^

There are also emerging data showcasing the potential benefits of guselkumab in PG treatment.^56^, ^57^, ^58^ Risankizumab, based on a few case studies, has similarly shown promise in alleviating PG.^59^, ^60^

##### Hidradenitis suppurativa

Ustekinumab is grounded in the hypothesis that the inflammatory processes in HS might be linked to disrupted Notch signaling.^17^, ^61^ It is postulated that anomalies in Notch signaling can cause aberrant differentiation and heightened destruction of outer hair root sheath cells. This process culminates in releasing keratins, which are believed to activate macrophages and dendritic cells by binding to toll‐like receptors. This activation cascades into the secretion of pro‐inflammatory cytokines IL‐23 and IL‐12, which further stimulate Th17 cells. These cells release additional cytokines, perpetuating the inflammatory cycle. By inhibiting both IL‐23 and IL‐12, ustekinumab may attenuate this cycle of immune disturbance. Although only a singular open‐label clinical trial has been conducted to assess the efficacy of ustekinumab in HS treatment, the preliminary results are encouraging. Patients demonstrated notable improvements in skin lesions, as measured by the sartorius score, and exhibited enhanced quality of life, as gauged by the DLQI. With its distinct inhibitory mechanism, ustekinumab might offer a fresh therapeutic avenue for HS, especially for those who do not respond to existing anti‐TNF treatments.

Guselkumab, an antibody specifically targeting the p19 subunit of IL‐23, has shown efficacy in treating severe HS at Hurley stages II/III or III.^62^, ^63^, ^64^ Several promising case reports and retrospective analyses have been presented, typically involving a dosing regimen of 100 mg at weeks 0 and 4, followed by 8‐week intervals.^62^, ^63^

### 2.2 Small molecules

#### 2.2.1 Janus kinase inhibitors

##### Pyoderma gangrenosum

The Janus kinase (JAK)/STAT signaling pathways play pivotal roles in numerous inflammatory skin conditions, and their dysregulation is evident in PG skin lesions. Notably, JAK‐1, JAK‐2, JAK‐3, and Signal transducer and activator of transcription (STAT) are markedly overexpressed in PG patients' skin relative to that of healthy individuals.^13^, ^26^, ^65^ Several JAK inhibitors have been explored for PG treatment, among them tofacitinib (targeting JAK‐1 and JAK‐3),^66^ ruxolitinib (specific to JAK‐1 and JAK‐2),^67^, ^68^ and baricitinib (inhibiting JAK‐1 and JAK‐2).^69^ There is empirical support, primarily from off‐label utilization, for the efficacy of these medications in PG, with particular emphasis on tofacitinib and ruxolitinib, as well as some evidence for baricitinib.^70^

##### Hidradenitis suppurativa

The JAK inhibitor upadacitinib has demonstrated efficacy in the treatment of HS, as evidenced by a retrospective cohort study indicating HiSCR50 and HiSCR75 achievements.^71^ Tofacitinib has also been documented in several case reports.^72^ Only one phase II study has been identified in the current literature that investigates the efficacy of the JAK 1 inhibitor INCB054707 for HS. Notably, this study, registered under ClinicalTrials.gov (NCT03569371 and NCT03607487), yielded positive outcomes.^73^

Additional research efforts, both completed and ongoing, delve into the potential of JAK inhibitors for HS management. Notably, a phase II randomized controlled trial (RCT) involving 192 patients (NCT04092452) is examining three kinase inhibitors: PF‐06650833 (an Irak4 inhibitor), brepocitinib (a Tyk2/JAK1 inhibitor), and ropsacitinib (a Tyk2 inhibitor). Concurrently, a phase I trial (NCT04772885) is in progress with 124 healthy participants, focusing on KT‐474, another Irak4 inhibitor.^74^, ^75^

#### 2.2.2 Phosphodiesterase 4 inhibitors: Apremilast

##### Pyoderma gangrenosum

Phosphodiesterase 4 (PDE4) is an enzyme responsible for hydrolyzing cyclic adenosine monophosphate (cAMP), an essential secondary messenger across all cell types. Apremilast, an orally taken small molecule, selectively targets and inhibits PDE4. This action, in turn, modulates the expression of various inflammatory mediators, including IL‐2, IL‐6, and TNFα.^76^, ^77^ Notably, TNFα and IL‐6, integral components of the cAMP signaling pathway, exhibit dysregulation in PG lesions. Several studies have reported the potential of apremilast as a monotherapy for stubborn PG,^78^, ^79^ while others have explored its combined efficacy with vedolizumab, a monoclonal antibody that targets the α4β7 integrin.^80^

##### Hidradenitis suppurativa

In the context of HS, multiple inflammatory cell types have been identified as playing a role. Given this, there is a theoretical foundation for the potential benefits of apremilast in managing HS. Several case reports have highlighted the promise of apremilast as a sustained treatment option for HS, demonstrating enduring clinical efficacy.^76^, ^81^, ^82^ In one randomized controlled trial (RCT) involving apremilast administered at 30 mg twice daily, 20 patients were stratified in a 3:1 ratio. This resulted in 15 patients with mild‐to‐moderate HS receiving apremilast, while five were on a placebo. Notably, 53.3% of those on apremilast achieved HiSCR after 16 weeks, in contrast to none in the placebo group, although this finding hovered at the edge of statistical significance with a *P* value of 0.055.^74^, ^76^ In contrast to this, a meta‐analysis revealed that apremilast did not demonstrate significant efficacy when treating moderate‐to‐severe HS.^41^

## 3 CONCLUSION

This review evaluates both established and emerging immunotherapies for PG and HS, drawing primarily from clinical observations. Managing PG and HS remains a formidable challenge, with immunosuppressive treatments standing as the foundational therapeutic approach. A deeper comprehension of the molecular underpinnings of PG and HS promises to usher in innovative, targeted interventions.

Early diagnosis and intervention for PG and HS are crucial, not only to ensure effective treatment and enhance the patient's quality of life but also to counteract systemic inflammation, a phenomenon observed in conditions such as psoriasis and atopic dermatitis. While direct evidence for such systemic effects remains elusive in the context of PG and HS, the activation of white blood cells and ensuing cutaneous inflammation could potentially herald complications in the cardiovascular system and other organs.^83^, ^84^, ^85^, ^86^, ^87^, ^88^, ^89^, ^90^, ^91^ Given these potential risks, it is imperative to recognize the value of timely deployment of potent treatments, such as biologics and small molecules, to mitigate inflammation.

## CONFLICT OF INTEREST STATEMENT

Keiichi Yamanaka is an Editorial Board member of *Journal of Dermatology* and an author of this article. To minimize bias, he was excluded from all editorial decision‐making related to the acceptance of this article for publication.
